# Genomics Analysis to Identify Multiple Genetic Determinants That Drive the Global Transmission of the Pandemic ST95 Lineage of Extraintestinal Pathogenic *Escherichia coli* (ExPEC)

**DOI:** 10.3390/pathogens11121489

**Published:** 2022-12-07

**Authors:** Fufang Xia, Jinlong Cheng, Min Jiang, Zhongxing Wang, Zhe Wen, Min Wang, Jianluan Ren, Xiangkai Zhuge

**Affiliations:** 1 MOE Joint International Research Laboratory of Animal Health and Food Safety, College of Veterinary Medicine, Nanjing Agricultural University, Nanjing 210095, China; 2 Department of Nutrition and Food Hygiene, School of Public Health, Nantong University, Nantong 226019, China

**Keywords:** ST95 ExPEC, comparative genome analysis, genetic determinants, global transmission, zoonotic potential, rapid/specific detection

## Abstract

Extraintestinal pathogenic *Escherichia coli* (ExPEC) is a pathogen that causes host extraintestinal diseases. The ST95 *E. coli* lineage is one of the dominant ExPEC lineages in humans and poultry. In this study, we took advantage of extensive *E. coli* genomes available through public open-access databases to construct a detailed understanding of the phylogeny and evolution of ST95. We used a high variability of accessory genomes to highlight the diversity and dynamic traits of ST95. Isolates from diverse hosts and geographic sources were randomly located on the phylogenetic tree, which suggested that there is no host specificity for ST95. The time-scaled phylogeny showed that ST95 is an ancient and long-lasting lineage. The virulence genes, resistance genes, and pathogenicity islands (PAIs) were characterized in ST95 pan-genomes to provide novel insights into the pathogenicity and multidrug resistance (MDR) genotypes. We found that a pool of large plasmids drives virulence and MDR. Based on the unique genes in the ST95 pan-genome, we designed a novel multiplex PCR reaction to rapidly detect ST95. Overall, our study addressed a gap in the current understanding of ST95 ExPEC genomes, with significant implications for recognizing the success and spread of ST95.

## 1. Introduction

The preeminent Gram-negative bacteria, *Escherichia coli*, can cause diverse human and poultry diseases, which include both intestinal and extraintestinal infections. When *E. coli* causes disease outside the host intestinal tract, with few exceptions, it is considered to be extraintestinal pathogenic *E. coli* (ExPEC) [1]. In accordance with the updated Clermont genotyping method, *E. coli* strains are classified into eight phylogenetic groups, which include A, B1, B2, C, D, E, F, and clade I [2]. Most ExPEC isolates are assigned to the B2, D, and F groups [3,4]. Strains of ExPEC harbor a broad array of virulence factors (VFs), which facilitate colonization into diverse ecological niches, such as the bloodstream, meninges, respiratory, and urinary tracts. Based on the disease pathologies, ExPECs are generally divided into uropathogenic *E. coli* (UPEC), neonatal meningitis *E. coli* (NMEC), septicemic *E. coli*, and avian pathogenic *E. coli* (APEC) [5,6]. These infections lead to considerable morbidity and mortality, plus enormous medical and social costs [7]. There is high population diversity among ExPEC strains. A retrospective and comprehensive review of ExPEC lineages revealed that the vast majority of extraintestinal infections are caused by a limited subset of pathogenic ExPEC lineages, which are defined by multilocus sequence typing (MLST) and named as sequence types (ST), such as ST131, ST95, ST69, ST73, and ST648 [8]. The dominant STs are repeatedly reported to be responsible for 40% to 60% of *E. coli* urinary tract infections (UTIs) and bloodstream infections (BSIs) in various areas of the world [8,9]. Regardless of the host or infectious niche, ExPECs share a mostly overlapping population of pandemic STs [10]. Furthermore, due to the misuse of antibiotics, several pandemic lineages of ExPEC isolates tend to be both high in pathogenicity and multidrug resistance. There is still insufficient evidence to explain ExPEC’s success as an extraintestinal pathogen, and the genetic determinants that promote its persistence, adaptability, and predominance in the extraintestinal reservoirs are yet to be revealed.

One of the predominant ExPEC lineages, ST95, belongs to phylogroup B2, is a cosmopolitan lineage, and is well-recognized as highly pathogenic. It commonly appears as a leading causative agent of UTI, BSI, and NMEC in humans. The ExPEC strains in the ST95 lineage exhibit a lower incidence of antimicrobial resistance when compared with other pandemic lineages [11]. It is not clear why ST95 isolates are so prevalent among clinical ExPEC strains, despite the fact that they are drug-susceptible. It has been hypothesized that other factors, rather than multidrug resistance, facilitate their dissemination. However, the emergence of ST95 isolates that are not susceptible to several antibiotics, such as β-lactam drugs and colistin, has been reported [12,13]. The ST95 lineage is also a predominant cause of avian colibacillosis [14]. There is a close correlation between APECs and human ST95 ExPECs, which are generally recognized to hold zoonotic potential. Multiple published studies have suggested that poultry may act as a reservoir of pathogenic ST95 in humans [15,16]. Human- and avian-origin ST95 strains share similar virulence [17] and genetic features [18,19]. In addition, ST95 APEC strains can cause meningitis and UTIs in the rat model of human ExPEC infections, and certain human ExPEC isolates are also highly virulent in chicks. These isolates are able to cause mortality in chicken models of colibacillosis, which provides substantial evidence for the claim that APECs and ExPECs hold zoonotic potential [20,21]. Consequently, the zoonotic potential of certain ST95 isolates cannot be undervalued: APECs/ExPECs pose a serious threat to human and animal health.

Given the widespread adoption of rapid and affordable high-throughput DNA sequencing technologies, there is an ever-increasing number of bacterial whole-genome sequences being made available. More studies now focus on the analysis of bacterial population structure and pathogenesis at a whole-genome level. The combination of genome sequencing and bioinformatics-driven sequence data analysis has brought dramatic advances in our view of how bacteria evolve, spread, and interact with each other or their hosts [22]. There is a far better understanding of the genetic features that may cause ST95 to develop into pandemic clinical ExPEC isolates.

In this study, we conducted a large-scale whole-genome analysis of 1081 ST95 strains. The population structure, dynamic evolution, and genetic characteristics of ST95 were revealed by analyzing the ST95 pan-genome and investigating the presence of diverse plasmids and pathogenicity islands (PAIs). These results provided a genomic framework to develop a deeper and more comprehensive understanding of ST95 ExPEC. Moreover, a novel multiplex PCR method was designed to cost-effectively detect the ST95 isolates. This method was compared with MLST, which is the expensive and time-consuming gold standard for the identification of ST95. We validated the PCR method to accurately distinguish ST95 from non-ST95 *E. coli*. The ability to distinguish between the sequence types will be significant for molecular epidemiological studies and useful for improving ST95 ExPEC surveillance. This study will provide a theoretical basis for the promotion of pathogen identification and targeted therapies to prevent and treat diseases caused by ST95 ExPEC.

## 2. Materials and Methods

### 2.1. Dataset Collection for Comparative Genomics Analysis

A total of 1081 ST95 genomic sequences were downloaded from the Enterobase (https://enterobase.warwick.ac.uk/species/ecoli/search_strains?query=st_search, accessed on 12 November 2022) and NCBI (https://www.ncbi.nlm.nih.gov/genome, accessed on 12 November 2022) databases. Detailed information for the ST95 strains collected in this study is listed in Table S1.

### 2.2. Pan-Genome Analysis and Phylogenetic Analysis

The large-scale pan-genome of ST95 was identified using Roary3.7.0 [23] and a standalone pan-genome pipeline, which were used to create a substantial core genome alignment. Single-nucleotide polymorphism sites were used to generate a SNP alignment from the core genome alignment. A maximum-likelihood (ML) phylogeny was constructed from the alignments using RaxML, with a general time-reversible (GTR) gamma model of nucleotide substitution [24]. The Interactive Tree of Life (iTOL) online-based software was used to visualize and annotate the ML phylogenetic tree (https://itol.embl.de/, accessed on 12 November 2022).

Based on the presence or absence of accessory genes, a binary alignment was generated, and an accessory tree was built from the alignments. Using accessory gene content, through Bayesian clustering via Kpax2, the accessory genome matrix was created using Roary to detect clusters of ST95 strains [25]. Five independent runs were performed using varied starting setups under the default parameters and upper-limit values for the number of clusters in the 30 to 50 range. The log posterior scoring function of this method was applied to determine the optimum clusters. The ‘image (A)’ function in Matlab was used to generate a heatmap to illustrate the pairwise similarities of accessory genome content for ST95 strains. ‘A’ referred to an arbitrary square matrix.

### 2.3. In Silico Characterization of ST95 Genomes

In silico serotyping was performed using SerotypeFinder v2.0 [26], from the CGE web server, with a threshold of 85% identity and at least 60% HSP-Length (http://cge.cbs.dtu.dk/service/SerotypeFinder/, accessed on 12 November 2022). Subtyping of *fimH* was conducted with FimTyper [27], with a threshold of 90% identity and at least 60% HSP-Length (https://cge.cbs.dtu.dk/services/FimTyper/, accessed on 12 November 2022). Genotyping for *gyrA* and *parC* was conducted by clustering sequences obtained from Roary, using USEARCH [28]. The alleles of *gyrA* and *parC* were detected as described by Johnson et al. [29].

### 2.4. Time-Scale Phylogenetic Analysis

Temporal analysis of ST95 was performed on the SNP alignments, using BEAST. The generalized time-reversible (GTR) nucleotide substitute model was used [30]. Various combinations of the molecular clock model (a strict, lognormal relaxed, and exponential relaxed molecular clock) and variable population growth (constant size and Bayesian skyline) were compared to find the model with the best fit. Three replicates were used for each model to run convergence and mixing, and the effective sample size (ESS) was assessed using Tracer v1.7 [31]. The models that converged well and had ESS values over 200, for all parameters, were considered to be acceptable. Bayes factors (BF), calculated from the marginal likelihood estimation (MLE) using steppingstone and path samplings were used to estimate the most suitable model for the dataset [32]. Under the strict clock, the Bayesian skyline model was the best-fit model for ST95. A Maximum clade credibility (MCC) tree was generated using tree annotator, which is included in the BEAST package, with 10% burn-in. The Bayesian skyline plot was formed using Tracer v1.6.

### 2.5. Virulence Genes, Antimicrobial-Resistant Genes and Plasmid Replicon Analysis

In this study, we used open-access bioinformatic webtools from the CGE online platform (http://www.genomicepidemiology.org/, accessed on 12 November 2022). The identification of virulence genes was performed using VirulenceFinder [33], with a threshold of 90% identity and 60% query coverage (https://cge.cbs.dtu.dk/services/VirulenceFinder/, accessed on 12 September 2021). Resistance genes were identified by ResFinder [34], with an identity threshold of 90% and query coverage of 90% (https://cge.cbs.dtu.dk/services/ResFinder/, accessed on 12 November 2022). Plasmid replicon sequence analysis was conducted using PlasmidFinder [35], with a threshold of 90% identity and 90% query coverage (http://cge.cbs.dtu.dk/services/PlasmidFinder/, accessed on 12 November 2022). The heatmaps were generated using ggplot2 in the R package to shift gene presence or absence data into a color scale. A principal component analysis (PCA) was conducted using the ade4 package in the R software. Co-occurrence network analyses were performed using gephi0.9.2 (https://gephi.org/, accessed on 12 November 2022).

### 2.6. Plasmid Analysis

The following approach was used to carry out plasmid analysis [36]. All protein sequences in our genomes were queried against the NCBI plasmid database to identify plasmid-associated genes in ST95 strains. These genes were mapped to the ST95 genomes, from which we acquired the contigs that were associated with plasmids. Subsequently, BLAST was used to align these contigs against the plasmid database, and non-plasmid sequences were removed (contigs with less than 10kb matching bases, at least 60% coverage; contigs with more than 10kb matching bases, at least 40% coverage). The non-redundant contigs that contained plasmid fragments were compared with each other to find similar sequences, with 90% length coverage. The largest contigs were extracted as representative sequences. These were further queried against the plasmid database using BLAST to identify corresponding plasmids in the ST95 genomes.

### 2.7. Detection of Pathogenicity Islands (PAIs) in ST95 

The Pathogenicity Island Database (PAIDB) (http://www.paidb.re.kr/about_paidb.php, accessed on 12 November 2022) is a user-friendly web-based resource that is dedicated to providing comprehensive information on all annotated PAIs. It is broadly used for the detection of PAIs in sequenced genomes. The known PAIs, retrieved from PAIDBplus extra PAIs against genomes [37] as found in ST95 strains, were detected by BLAST. Only PAIs with hits of >50% identity were considered to be present.

### 2.8. Development and Validation of a Multiplex PCR Assay to Rapidly Distinguish ST95 APEC/ExPEC Isolates

The PCR primers were designed to target ST95-specific genes using Primer 5 software. After empirically optimizing the PCR conditions and evaluating several candidate primer pairs using diverse ST *E. coli* strains, the best primer pairs were combined in a multiplex PCR. Amplification was conducted in 20 μL of reaction mixture, which included 10 μL Green Taq Mix (Vazyme), 0.5 μL of each primer, 1.5 μL of DNA template, and 4.5 μL ddH_2_O. The cycling conditions were as follows: an initial denaturation for 5 min at 94 °C, 30 cycles of 94 °C for 30 s, 60 °C for 30 s, and 72 °C for 60 s, with a final extension step of 72℃ for 5 min, followed by a hold at 4 °C. The PCR products were loaded onto a 1% agarose gel with GelRed. After electrophoresis, the gels were photographed under UV light.

Validation of the multiplex PCR assay was performed using avian- or human-source *E. coli* isolates. Isolates were assigned to the phylogenetic groups by Clermont’s quadruplex multiplex PCR [38]. The MLST method was performed as previously described, using the MLST scheme for amplifying and sequencing the seven *E. coli* housekeeping genes. Gene sequences for the housekeeping genes were submitted to the MLST database (http://enterobase.warwick.ac.uk/species/ecoli/allele_st_search, accessed on 12 November 2022), and the *E. coli* STs were matched [11].

## 3. Results

### 3.1. Pan-Genome Analysis and Phylogeny of ST95 ExPEC

We retrieved a total of 1081 publicly available whole genomes from ST95 strains from the NCBI and Enterobase databases. These included 788 human and 135 avian strains (see Table S1 in Supplemental Materials for details), which originated from different times, diverse hosts, and various countries. The use of diverse strains improved the geographic and temporal diversity in the comparative genome analysis. We utilized these genomes to identify the ST95 pan-genome. The pan-genome for our collection was estimated to have 30,297 genes, which included 2964 core genes that were shared by >99% of the strains, 687 soft core genes that were shared by 95% to 99% of strains, 2021 shell genes that were found in 15% to 95% of ST95 strains, and 24,625 cloud genes that were found in <15% of the strains. These data showed considerable genomic diversity and high genomic plasticity among the ST95 strains.

**Figure 1:**
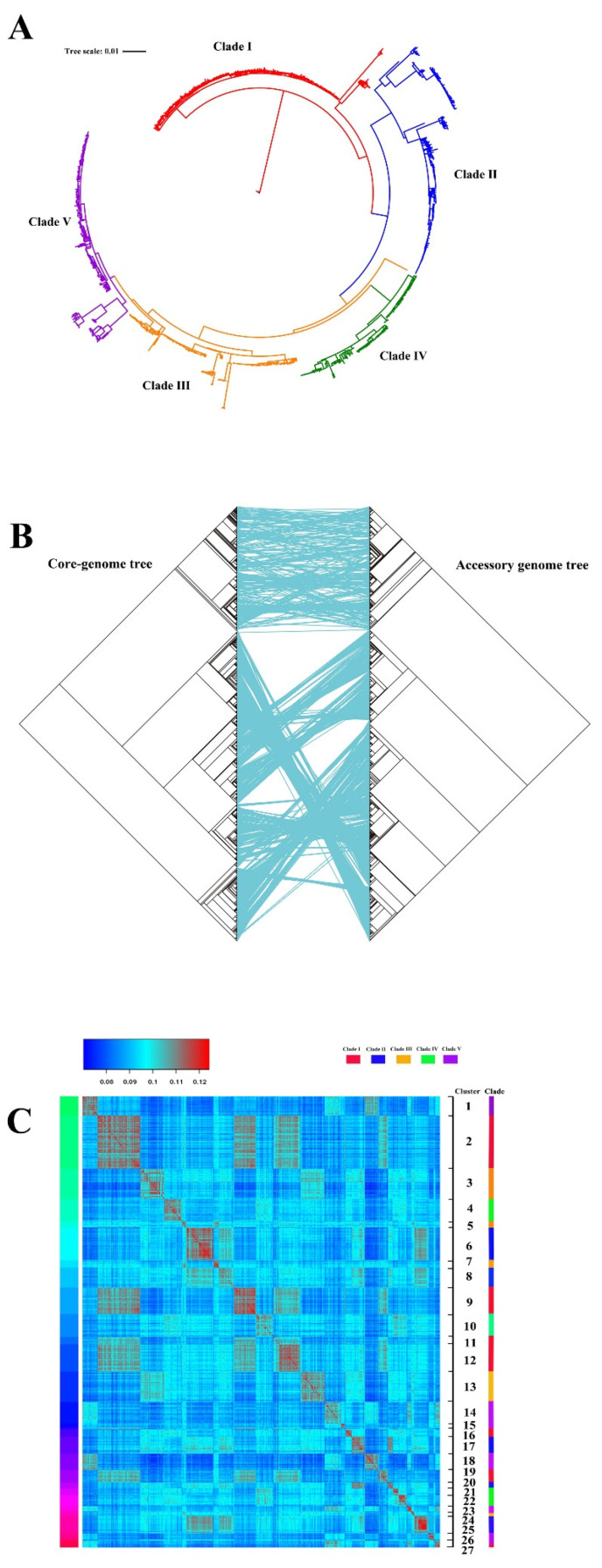
Phylogeny of ST95 isolates. (**A**) Phylogenetic tree of ST95 isolates constructed using a SNP alignment of the core genome. Branches of the tree are colored according to the clade, where red is Clade Ⅰ, blue is Clade Ⅱ, green is Clade Ⅳ, yellow is Clade Ⅲ, and purple is Clade Ⅴ. The tree scale bar denotes substitutions per variable site. Branch support was performed with 1000 bootstrap replicates. (**B**) Comparison between the core genome tree (left) and the accessory tree (right), constructed from binary presence/absence data of the accessory genes. The identical tree tips are connected by lines. (**C**) Diagrammatic representation of the clustering of ST95 isolates, according to their accessory gene content by pairwise comparison of the accessory gene content of all 1081 genomes. The heatmap shows the similarity of accessory genomes between strains, as formed using the BLAST score, with red representing 100% and dark blue representing 0%. The color bands on the left and the scale on the right indicate the accessory genome clusters. The color coding on the right side indicates the clade to which the strain belongs.

To determine the evolutionary relationships between these strains, we constructed a maximum-likelihood (ML) phylogenetic tree based on core-genome single-nucleotide polymorphisms (SNPs) (Figure 1A). The tree clearly demonstrated a highly structured population with genetic diversity, which was distinctly divided into five clades, designated Clade Ⅰ to Clade Ⅴ (Figure 1A). Table S2 displays the assignment of all strains in each clade. The distribution of these ST95 strains was as follows: Clade Ⅰ (30.5%), Clade Ⅱ (20.2%), Clade Ⅲ (17.0%), Clade Ⅳ (14.3%), and Clade Ⅴ (18.0%). A phylogenetic tree of the accessory genome was also created using the presence or absence of genes in the accessory genome. A comparison between the core genome and accessory genome trees indicated that ST95 strains situated at a similar branch of the core genome tree were also accessible at a parallel branch of the accessory genome tree (Figure 1B). Each clade began with a specific set of accessory genes that had remained virtually unchanged over subsequent evolutionary history. Moreover, by means of the Bayesian clustering analysis tool, K-Pax2 [25], the accessory genome was clustered based on the accessory gene content, which generated 25 diverse clusters of strains (Figure 1C and Table S2). Cluster analysis on a pairwise comparison of the ST95 accessory genomes indicated that there were obvious differences in ST95 accessory genomes across the five clades. The ST95 strains that belonged to the same core genome clade varied in accessory genome composition. The high variability of accessory genes emphasized the diversity of the genomes from ST95 ExPEC.

### 3.2. Genotype Characterization of ST95 ExPEC

**Figure 2:**
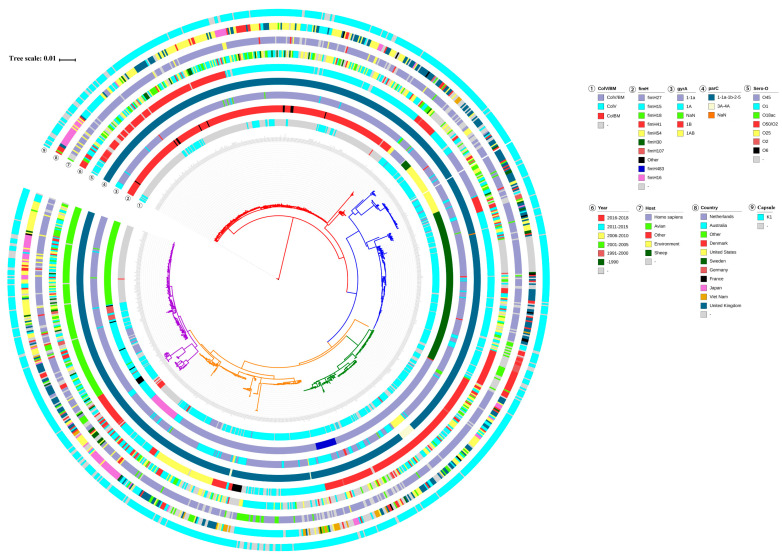
Genotype characterization of ST95 ExPEC strains. The phylogenetic tree in the center is identical to that in Figure 1A. The concentric colored rings, from innermost to outermost, indicate the carriage of ColV/ColBM types, *fimH* alleles, *gyrA* genotypes, *parC* genotypes, O serotypes, collection year, host source, country of origin, and the presence of capsular K1.

We mapped the geographic and host origin of ST95 strains onto the phylogenetic tree and found clades without significant associations to host or country (Figure 2). The ST95 strains from different geographical regions were diversely located on different branches of this tree. Similarly, there was no major clustering of host sources on the tree, which indicted that ST95 underwent frequent inter-species transmission and maintained its zoonotic potential. In addition, almost all ST95 strains possessed the K1 capsule. Although the serogroups of ST95 were diverse, the dominant serogroups represented were O1 (37.5%), O50/O2 (31.7%), and O18ac (14.8%).

The ColV/ColBM plasmids have been identified as a feature of APEC/ExPEC isolates from animals. However, they are rarely observed in ExPEC strains of human origin [39,40]. The ColV/ColBM plasmids may therefore be employed as a definitive feature to reveal zoonotic transmission from poultry to humans. Here, we mapped the presence of ColV/ColBM plasmids onto the phylogenetic tree (Figure 2). It was evident that the majority of ST95 strains appeared to contain ColV/ColBM plasmids. The level of ColV/ColBM plasmids among human ST95 strains was not as low as that reported in the above referenced reports, which suggested that the ColV/ColBM plasmids may be disseminated from poultry to humans. We also found that isolates in Clade Ⅰ and Ⅴ displayed lower levels of ColV/ColBM plasmids than isolates that belonged to Clade Ⅱ, Ⅲ, or Ⅳ.

We also mapped *gyrA* genotypes, *parC* genotypes, and *fimH* variants across the ST95 collection (Figure 2). The sequence analysis of the *gyrA* and *parC* genes in 1081 ST95 strains revealed five *gyrA* alleles and six *parC* alleles. However, most strains contained allele combinations, of which the most common were *gyrA*1-1a and *parC*1-1a-1b-2-5, that were not associated with fluoroquinolone resistance. It is worth noting that *gyrA*-1AB and *parC*3A-4A occurred simultaneously in 16 strains (1.4%), and this combination conferred fluoroquinolone resistance onto the strains. The *fimH* gene plays a vital role in ExPEC urothelial adhesion and has been recognized as a valuable genetic marker to improve the resolution of MLST for ExPEC strains [41,42]. Among our collection, the most prevalent types were *fimH*41 (27.5%) and *fimH*27 (27.1%), followed by *fimH*30 (15.1%) and *fimH*15 (8.3%). Of significant interest was a link between *fimH* variants and the clades in the core genome phylogenetic tree. The high level of allelic diversity of *fimH* also contributed to the high level of genetic diversification of the ST95 lineage. Strains that belonged to Clade Ⅱ were characterized by *fimH54* and *fimH30*, while 90.2% of strains in Clade Ⅰ carried *fimH41*. Clade Ⅳ comprised the ST95 strains that encoded *fimH27* and *fimH*438. All the Clade Ⅲ strains encoded *fimH27*, while Clade Ⅴ contained genes that encoded several *fimH* alleles, which included *fimH15*, *fimH18*, *fimH41,* and *fimH107*.

### 3.3. Time-Scale Emergence of ST95 ExPEC

**Figure 3:**
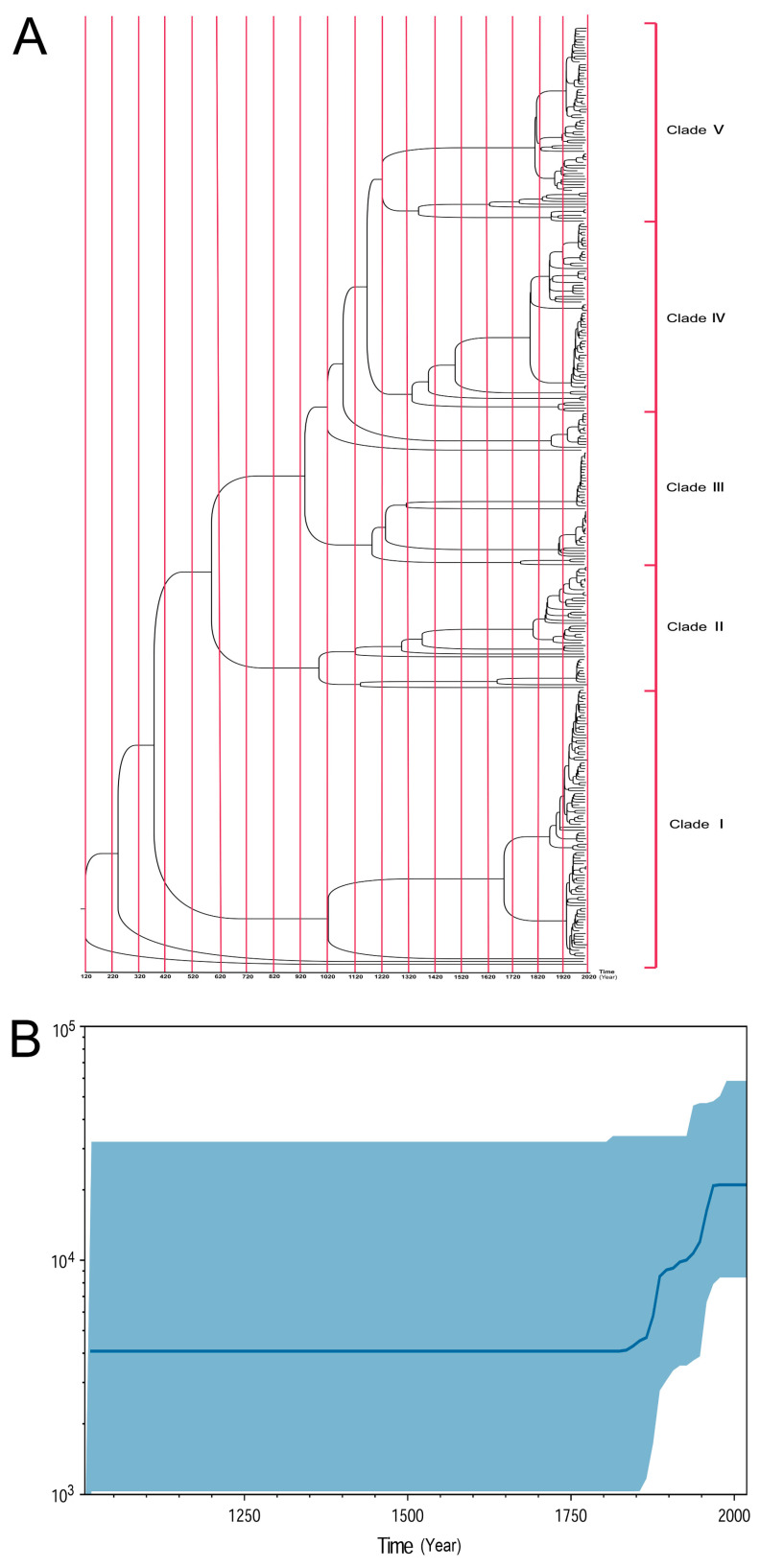
Temporal analysis of ST95 ExPEC isolates by BEAST. (**A**) Time-scale Bayesian phylogenetic tree reconstructed using SNP alignments of 336 ST95 isolates. Time periods and age of clades can be inferred using the timeline at the bottom. Figure S1 in the Supplementary Materials contains detailed information on ST95 strains. (**B**) A Bayesian skyline plot for the estimated population size of ST95. The blue curve refers to the effective population size, and the blue shaded range shows the change in the 95% confidence interval. The *x*-axis gives time in years, and the *y*-axis gives the population size.

To identify the evolution of ST95 ExPEC and estimate the date of the emergence of the five distinct ST95 clades, a time-scaled phylogenetic tree was generated using Bayesian evolutionary analysis by sampling trees (BEAST) (Figure 3A) [30]. This scaled phylogeny showed that the most recent common ancestor (MRCA) for ST95 was dated to approximately 1900 years ago, and the emergence of an avian-source ExPEC isolate, ESC_RA4279AA_AS, was close to the ST95 MRCA. The time-scaled phylogeny indicated that the strains in Clade Ⅰ diverged from the ESC_BA6230AA_AS ExPEC strain approximately 1775 years ago (95% highest probability density, HPD) (Figure 3A). The separation between Clade Ⅰ and Clade Ⅱ was indicated to have occurred approximately 1640 years ago (95% HPD). The split between Clade Ⅱ and Clade Ⅲ was estimated to have occurred approximately 1425 years ago (95% HPD). The separation between Clade Ⅲ and Clade Ⅴ dated back approximately 940 years (95% HPD) and Clade Ⅳ diverged from Clade Ⅴ around 860 years ago (95% HPD). Time evolution indicated that the ST95 strains in each clade have evolved several subtypes in the past two hundred years. The evolutionary rate of ST95 was estimated using BEAST, and the substitution rate was predicted to be 7.67 × 10^−7^ mutations per site per year.

We further employed the Bayesian skyline model to investigate the effective population size of ST95 in the past. A Bayesian skyline plot demonstrated three sequential increases in the predicted effective size of the ST95 population. The first rapid increase started in the early 1840s, followed by a steady growth that happened around the 1890s. The last enlargement occurred sharply, close to 1950 (Figure 3B). After the 1970s, the population size of ST95 leveled out. The results were indicative of the fact that ST95 is an old and long-standing *E. coli* lineage, with a long evolutionary history. It has successfully persisted for a long time and evolved in parallel in both humans and poultry.

### 3.4. Carriage of Virulence Genes in ST95 ExPEC

**Figure 4:**
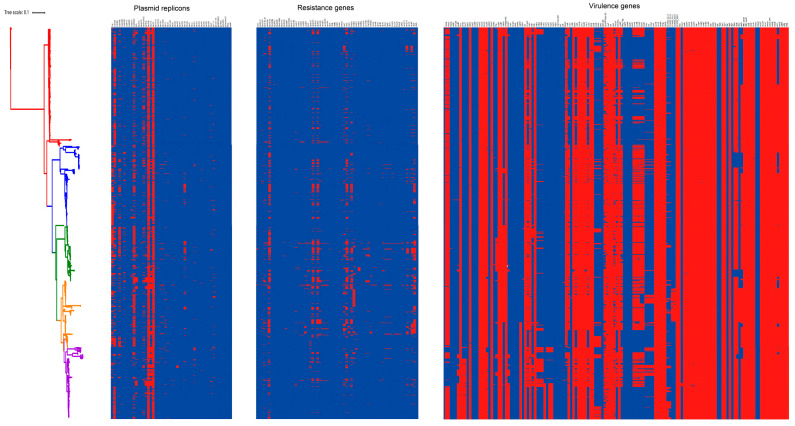
Heatmap showing the presence or absence of plasmid replicons, resistant genes, and virulence genes across all 1081 ST95 isolates. On the left is the ML tree from Figure 1A, shown as a vertical clade diagram. The columns adjacent to the phylogenetic tree, from left to right, represent the presence or absence of plasmid replicons, resistant genes, and virulence genes. The red and blue indicate the presence or absence of each gene, respectively. The detailed distribution and the full list of examined genes in each category is provided in Supplementary Table S3.

Given that previous studies have indicated that ST95 is a pandemic lineage that leads to community-onset infections, investigation of the virulence genes carried by ST95 isolates [13] is of prime importance. Using the Virulence Finder database, we analyzed the distribution of 136 virulence genes to obtain a good command of the extent of within-clade diversity and the high virulence potential of ST95. The ST95 isolates showed a high frequency of ExPEC-associated virulence genes, and there was a huge diversity in ST95 virulence gene profiles (Figure 4). Table S3 summarizes the detailed distribution of virulence genes among ST95 isolates. Each strain often carried several virulence genes from specific functional categories, which included adhesins, toxins, invasins, iron acquisition systems, and protectins.

**Figure 5:**
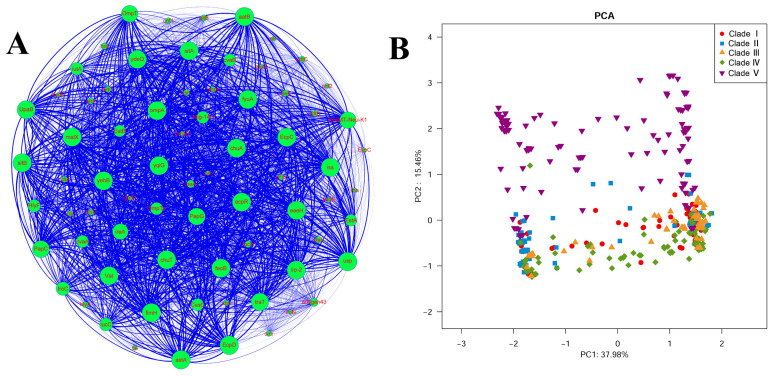
Co-occurrence analysis and PCA analysis of virulence genes in ST95 strains. (**A**) Co-occurrence analysis of virulence genes in ST95 isolates. Nodes represent virulence genes. The number of strains carrying the gene is shown by node size. The blue lines linking the nodes indicate co-occurrence, and the line widths indicate the number of co-occurrences. (B) PCA analysis of the profiles of virulence genes. A specific and colored shape represents isolates that belong to each clade (Clade Ⅰ: red circles; Clade Ⅱ: blue squares; Clade Ⅲ: yellow triangles; Clade Ⅳ: green rhombuses; Clade Ⅴ: purple inverted triangles).

To visualize the frequency of virulence genes in these strains, co-occurrence of the prevalent virulence genes was further analyzed by a network analysis (Figure 5A). Several genes (*fimH*, *papG*, and *papC*) that encode fimbriae and additional adhesins, which are responsible for colonizing the host, were detected at a high frequency. Similarly, genes that are involved in iron-uptake systems were widespread in ST95. The most common iron-acquisition genes were *feoB* (a ferrous iron transporter) and *chuA* (an outer membrane hemin receptor), which were present in all isolates, followed by *fyuA* and *irp2* (yersiniabactin), *sitA*, and *sitB*. The *iutA*, *iucC* (an aerobactin operon), *ireA* (iron-regulated virulence gene), and *iroC* (salmochelin) genes were less common, found in 58.5%, 58.6%, 79.9%, and 65% of strains, respectively.

The distributions of the five ExPEC-specific protection genes, *iss*, *traT*, *ompT*, *kfiC*-K5, and *kpsMT*-Neu-K1, were also determined. Almost all strains carried the serum survival gene, *iss* (99.2%). Approximately 99.2%, 91.8%, and 83.8% of strains carried *traT*, *ompT*, and *kpsMT*-Neu-K1, respectively. Toxin-related genes were also investigated, and the most frequently identified were *vat* (vacuolating autotransporter toxin) and *astA* (EAST1 toxin), which were carried by 97.2% and 99.9% of strains, respectively. Approximately 56.8% of strains carried *hlyF*, while *hlyA* and *hlyD* were specifically detected in 10.4% of isolates. Other toxin genes (*tsh*, *cnf2*, *cdtA*, *cdtB*, and *cdtC*) were identified in <12% of ST95 strains. The invasion-related gene, *IbeA*, which is an important virulence factor of ExPEC and is responsible for neonatal meningitis in humans, was detected in 21.5% of strains. The distribution of the ColV plasmid trait genes was also determined. Approximately 55.5% of isolates were positive for *cvaA* and *cvaB*, while 54.6% were positive for *cvaC*.

To further elucidate the relationship between the virulence gene profiles and the population structure of ST95, we conducted a principal components analysis (PCA) using the matrix of virulence gene presence or absence. We further mapped the five clades onto the PCA data (Figure 5B). The five clades could be partially separated via the first two components, which accounted for 53.44% of the overall variation. The presence of multiple virulence genes differed among ST95 strains from different clades. The *sfaS*/*focD* (S and F1C fimbriae), *ibeA*, and toxin genes (*cnf1*, *cnf2*, *hlyA*, and *hlyD*) were exclusively detected in Clade Ⅴ strains. Nine other genes, which included *cdtABC* (cytolethal distending toxin), *eitABC*, and *hek*/*hra* (heat-resistant agglutinin), also had a higher prevalence in Clade Ⅴ. The *ireA* gene was detected in significantly fewer strains in Clade Ⅴ when compared with other clades. The distribution of the genes that encode adhesins, invasions, and toxins in the Clade Ⅴ strains suggested a high degree of virulence potential in the Clade Ⅴ isolates. Clade Ⅰ displayed a low level of ColV/BM plasmid-related genes, such as *hlyF*, *ompTp*, *mig-14p*, and *iutA*. When compared with Clade Ⅰ and Clade Ⅴ strains, strains that belonged to Clades Ⅱ, Ⅲ, and Ⅳ shared similar virulence gene profiles. However, there were some notable differences. There was a higher presence of the autotransporter adhesin gene, *aatA,* in Clade Ⅲ strains. The autotransporter antigen 43 gene was significantly more prevalent in Clades Ⅲ and Ⅳ and was detected in all Clade Ⅲ strains, while it was not common in the strains from Clades Ⅰ, Ⅱ, and Ⅴ.

### 3.5. Carriage of Resistance Genes in ST95 ExPEC

**Figure 6:**
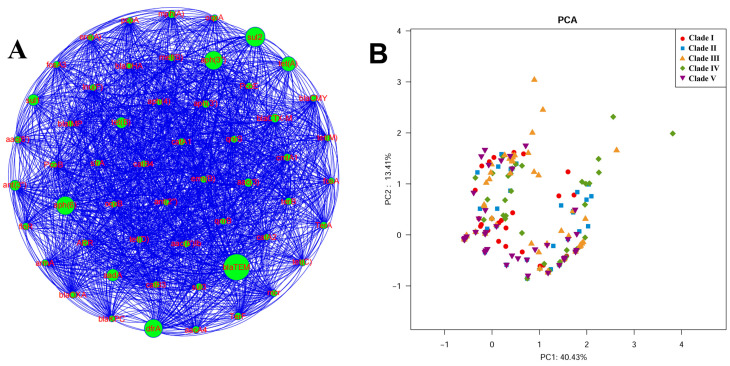
Co-occurrence analysis and PCA analysis of resistance genes in ST95 isolates. (**A**) Co-occurrence analysis of resistance genes in ST95 strains. Nodes represent resistance genes. (**B**) PCA analysis of the profiles of resistance genes. A specific and colored shape represents isolates that belong to each clade (Clade Ⅰ: red circles; Clade Ⅱ: blue squares; Clade Ⅲ: yellow triangles; Clade Ⅳ: green rhombuses; Clade Ⅴ: purple inverted triangles).

Given the clinical significance of antimicrobial resistance in ExPEC, we analyzed the resistance gene content of the ST95 strains. A heatmap showed the distribution of resistance genes among the strains (Figure 5). We observed that ST95 isolates demonstrated a low frequency of antibiotic resistance genes when compared with the virulence gene profile. Detailed information about resistance genes identified in ST95 strains is given in Table S3. Approximately 49% of ST95 strains carried resistance genes. Co-occurrence of the prevalent resistance genes was analyzed by a network analysis (Figure 6A).

The aminopenicillin resistance *β*-lactamase gene, *bla*_TEM-1_, found in 34.7% of ST95 strains, was the most common acquired resistance gene. Other genes that encode *β*-lactamases were detected at a relatively low frequency. The *bla*_CTX-M-1_ genotype is one of the most common genotypes of *E. coli* that produce extended-spectrum *β*-Lactamase (ESBLs), which are responsible for resistance to cephalosporin antibiotics. However, it was detected in just 4.3% of ST95 strains (47/1081). The presence of non-lactamase resistance genes was also investigated. Of the genes that encode sulfonamide resistance (*sul1*, *sul2*, and *sul3*), the *sul2* gene was found in 22.8% of ST95 strains and was more prevalent than *sul1* and *sul2* (8.6% and 1.5%, respectively). The *DfrA* gene confers trimethoprim resistance and occurred in 20.3% of ST95 strains. The *aph(3’’)* and *aph(6)* genes, which are types of aminoglycoside resistance genes, were detected in 21.2% and 21.1% of ST95 strains, respectively. Other aminoglycoside resistance genes, such as *aph(6’)-Ib*, *aph(3’)*, and *aph(6’)*, were found at a low frequency (<5% prevalence). The *tet(A)* gene, which was detected in 14.3% of strains, was the most predominant tetracycline resistance gene in ST95. The *AadA* gene confers streptomycin resistance and was detected in 10.1% of isolates, while two isolates contained *strA*. Macrolide resistance genes, such as *ere(A)*, *erm(B)*, *mph(A)*, and *mph(B)*, were detected in 36 isolates. Chloramphenicol resistance genes (*cmlA*, *catA*, *catB*, and *floR*) and fosfomycin resistance genes (*fosA* and *fosA3*) were very rare (<2% prevalence). Plasmid-mediated quinolone resistance (PMQR) genes, which included *oqxA*, *oqxB*, *qnrB*, and *qnrS*, were also rarely detected (<2% prevalence). Four isolates were found to carry the *mcr-1* gene, which is associated with resistance to colistin.

We determined whether the resistance gene profiles were correlated to the population structure of ST95. We conducted a PCA analysis, and a variance of 45.5% was captured in the resistance gene profiles (Figure 6B). While there was no significant difference in the content of resistance genes among strains from different clades, the strains in Clades Ⅲ and Ⅳ were more likely to carry more resistance genes.

### 3.6. The Diversity of Plasmid Replicons in ST95 ExPEC

**Figure 7:**
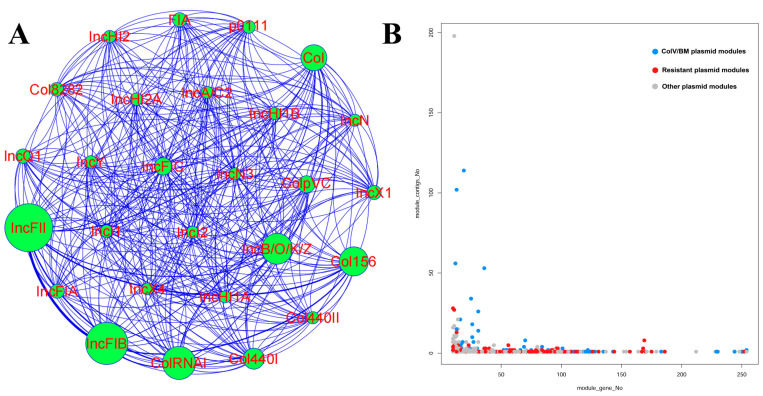
Co-occurrence analysis of the plasmid replicons and the presence of the plasmid modules in the ST95 collection. (**A**) Co-occurrence analysis of plasmid replicons in ST95 isolates. Nodes represent plasmid replicons. (**B**) Frequency distribution of the plasmid modules among ST95 isolates. The modules that carried 10 or more plasmid genes were mapped along the *x*-axis. The *y*-axis indicates the frequency distribution of the modules in ST95 strains. Red circles, blue circles, and grey circles represent modules containing resistance plasmid genes, modules containing ColV/BM plasmid genes, and modules containing unknown plasmid genes, respectively.

The Plasmidfinder database was used to identify the distribution of 50 plasmid replicons among the ST95 strains (Table S3). The heatmap (Figure 5) demonstrated a high diversity of plasmid replicon profiles and various combinations of plasmid replicon types in the ST95 strains. Only 2.7% of ST95 strains failed to carry any replicons. A network analysis was also performed to identify co-occurrence of the common plasmid replicons in ST95 strains (Figure 7A).

The most common plasmid replicons were IncFII and IncFIB, which were present in 92.3% and 90.9% of our ST95 collection, respectively. They were also frequently discovered together in the same isolates (89.5%). The IncFII and IncFIB replicons were correlated with large conjugative virulence plasmids. We also identified small col-like plasmid replicons, most of which were detected in isolates that carried IncFII and IncFIB replicons. This raised the possibility of the presence of large IncFII–IncFIB hybrid ColV-like virulence plasmids. The Col RNAI and Col156 replicons were also frequently identified in 43.3% and 44.3% of ST95 isolates, respectively. The Col156 plasmid was previously recognized as a vital ARG transmitter [43]. The common replicon combinations were IncFII–IncFIB–Col156 (42.4%) and IncFII–IncFIB–ColRNAI (39.8%). Approximately 26.9%, 18.8%, and 18.5% of ST95 strains contained the Col, Col 1440Ⅰ, and Inc B/O/K/Z replicons.

### 3.7. The Diversity of Mapped Plasmids within the ST95 Pan-Genome

Large plasmids are significant agents of horizontal gene transfer and harbor genes related to virulence and antibiotic resistance. They also promote bacterial adaptation to environmental changes. To determine the plasmid content of our ST95 collection, we initially queried all genes in the pan-genomes of the ST95 strains against the plasmid database to identify all plasmid-related genes. The genes that matched plasmid sequences were then mapped against the genome of each isolate. We found 978 modules that contained 10 or more plasmid genes, with the number of plasmid genes per module ranging from 10 to 254 (Table S4). We further assessed these modules against known plasmids from the plasmid database to increase the amount of information on the plasmids present in the ST95 strains. The frequency distribution of modules is shown in Figure 7B. Given the wide variety of module lengths and the number of strains that contained these modules, horizontal gene transfer appeared to play a significant role in the evolution of the ST95 genome. A number of types of plasmid that were related to virulence and antimicrobial resistance were detected among the ST95 isolates (Table S4).

The mapped ColV-like plasmid sequences that had a high frequency in the ST95 strains included plasmid pECOS88, plasmid pJIE186-2 from the EC958 ST131 strain, plasmid pC59-153, plasmid pAPEC-O78-ColV, and MS7163 plasmid pMS7163A (Table S4). These data imply that ColV-like large plasmids may undertake a critical role in shaping the development and evolution of the ST95 clades. The ColBM plasmids, such as plasmid pH17-5, also were mapped to the pan-genome of the ST95 strains. We further found other types of diverse virulence plasmids, such as plasmid pCFSAN029787_01, which contains T3SS, and plasmid pO26-Vir, which possesses multiple virulence genes such as *espP*, *katP*, *toxB*, and the *hly* gene cluster. The high prevalence of these virulence-associated plasmids acts to increase the virulence potential of ST95. Virulence plasmid pSF-166-1 was mapped to several strains in Clade Ⅰ and Clade Ⅴ and shared a high sequence similarity to plasmids pRS218, pUTI89, and pEC14_114 [44]. Plasmid pCVM29188_146, which is a resistance plasmid that was originally discovered in a poultry-derived *Salmonella enterica* strain and shares a highly conserved plasmid backbone with pAPEC-O1-ColBM and pAPEC-O2-ColV [45], was also detected in avian-derived and human-derived ST95 isolates. This indicated the transfer of large plasmids between poultry- and human-origin *E. coli* or between different species of bacteria.

Several mapped plasmids carried diverse types of resistance genes among the ST95 strains. There was a high prevalence of plasmids that carried the *bla*_TEM-1_ gene, such as plasmid pECAZ147_1, plasmid pPA45B, and plasmid pM160133_p2. This could explain the widespread presence of *bla*_TEM-1_ gene in ST95 strains (Table S4). The presence of plasmids that simultaneously carried virulence genes and antibiotic resistance genes, such as D6 plasmid A, UPEC 26-1 plasmid unnamed1, and plasmid pM160133_p2, was of great concern. Overall, the plasmids of ST95 were highly diverse, and there was no plasmid that was significantly clustered in a particular clade, which implied that there were no strong clade specific adaptations.

### 3.8. The Distribution of Genomic Islands among ST95 ExPEC Strains

Genomic islands are horizontally acquired DNA regions that are characterized by association with tRNA-encoding genes and a G+C content that is distinct from that of the core genome [46]. The specific features of PAIs and the accessibility of genome data provide viable methods for the identification of PAIs. To identify the presence of ExPEC-associated PAIs in ST95, we compared the genomes of the ST95 strains with 31 known PAI sequences, using BLAST [36]. The presence of PAIs among ST95 is shown in Table S5. As expected, ST95 strains carried multiple PAIs, most of which encoded ExPEC-related virulence genes and metabolism-related genes.

Almost any ST95 strain can possess the *Vat*-encoding PAI, which has previously been found in the APEC O1 genome and is situated close to the *thrW* tRNA gene. The *Vat* gene is involved in the pathogenicity of APEC/ExPEC strains [47]. Most of the strains in our collection were also found to harbor AGI-3, PAI-I (APEC-O1), PAI-III 536, and PAI-I AL862. The AGI-3 PAI is a *selC*-related genomic island that is involved in carbohydrate uptake and virulence. The PAI-I (APEC-O1) was first identified in APEC O1:K1 and contains the complete *pap* operon that carries the P pilus, the *ireA* gene, *kps* gene cluster, and the *tia* invasion gene [48]. The PAI-III 536 is bordered by the tRNA-encoding gene, *thrW,* and contains genes that encode S fimbrial, HmuR-like heme receptor, salmochelin, and a TSH-like hemoglobin protease [49]. The PAI-I AL862 harbors virulence factor AfaE-VIII adhesin, ribose metabolism, and the PTS system [50]. Of significant interest was that two genomic islands were exclusively detected in Clade Ⅴ strains. One was PAI-I 536, which was identified in 96 strains and is associated with the tRNA-encoding gene, *selC*. Several virulence factors, such as α-haemolysin, CS12 fimbriae, and F17-like fimbrial adhesin, were located on PAI-I 536. The other Clade V genomic island was the GimA island, which was conserved across all Clade Ⅴ strains. It is situated between *yjiD* and *yjiE* and contains the *ibeA* genes that are responsible for neonatal meningitis in humans [51]. Four genomic islands (GI-2, GI-7, GI-12, and GI-16) have been identified in the IMT5155 APEC O2:K1 strain [19]. However, some PAI sequences were not found in ST95 strains. These included the espC PAI, which was originally reported in enteropathogenic *E. coli* and encodes enterotoxin and ETT2, which are involved in the type III secretion system.

### 3.9. Rapid and Specific Detection of ST95 APEC/ExPEC Isolates

**Figure 8:**
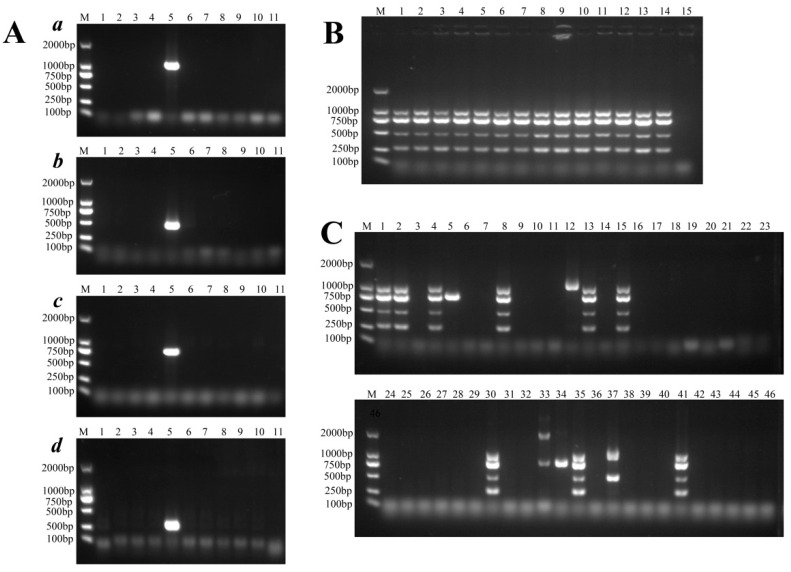
Rapid and specific identification of ST95 APEC/ExPEC isolates by PCR analysis. (**A**) Detection of putative ST95-specific genes in ExPEC strains by PCR. To confirm that genes (*a*, *sfmH*; *b*, *group4861*; *c*, *group8607*; *d*, *group9754*) are specific to ST95 strains, PCR assays were performed with ExPEC strains that belonged to different STs. M, DL 2000bp DNA Marker; Lanes 1 to 10 correspond to ST354, ST48, ST155, ST359, ST95, ST313, ST131, ST602, ST101, and ST2732 strains, respectively. Lane 11, no-template control. (**B**) Agarose gel electrophoresis of multiplex PCR products amplified from ST95 strains, using the developed multiplex PCR method, based on primers that detect *sfmH*, group8742, group 9754, and group8607. Lane M, 2000bp ladder. Lanes 1 to 14 are ST95 strains. Lane 15, no-template control. (**C**) Multiplex PCR for the detection of human- and poultry-source ST95 APEC/ExPEC isolates. The strain with four simultaneously amplified bands was identified as belonging to ST95. (**A**) Lane M, DL 2000bp DNA Marker; Lane 1, 2, 4, 8, 13, 15, 30, 35, and 41 are ST95 isolates. Other lanes are non-ST95 strains. Lanes 23 and 46, no-template control.

In a previous study, we collected a number of other ST *E. coli* genomes (such as ST117) to identify their pan-genomes [36]. We identified unique ST95 genes that could be used to develop a multiplex PCR method to rapidly screen ST95 clinical isolates. We compared the genes identified in the ST95 pan-genome with other STs and found that 2133 genes were unique to ST95 strains. Detailed information for these genes is listed in Table S6. We aligned the gene sequences to the reads from 134 published gut metagenomes [36], which contained the genomes of pandemic STs, to confirm whether they are also present in other *E. coli* and bacterial species (Table S7). Several genes were shown to have a high presence in the metagenomes that contained ST95 genomes but a very low prevalence in the non-ST95 gut metagenomes (Table S7). We further selected ten genes that occurred at high frequency in ST95 genomes. These were *sfmH*, *group4861*, *group8742*, *group9754*, *group8607*, *group13698*, *vgrG13*, *cas3*, *group9860*, and *group1782*. These unique genes were used to design ten pairs of primers to specifically detect ST95 and other ST ExPEC strains in a multiplex PCR. Finally, four unique genes (*sfmH*, *group4861*, *group8607*, and *group 9754*) were chosen for further PCR development based on their high degree of conservation and specificity (Figure 8A). As shown in Figure 8B, the multiplex PCR was used to detect the unique genes in ST95 strains. Primer sequences and product sizes are given in Table 1.

To validate the accuracy of the multiplex PCR method, we tested 1218 *E. coli* isolates of unknown STs from humans (*n* = 725) and poultry (*n* = 493) using multiplex PCR. The partial results of the multiplex PCR assay are shown in Figure 8C. A total of 191 isolates from humans (*n* = 127) and poultry (*n* = 64) yielded four products of the expected sizes. The strains were assigned to STs using the Achtmann MLST scheme. The isolates that produced four products were identified as ST95, while strains that produced one or two bands were identified as other STs, such as ST73 and ST1193.

To evaluate the specificity and sensitivity of this method, we investigated the examined isolates. The 1218 *E. coli* isolates were genotyped for phylogroups. Those from humans (*n* = 326) and poultry (*n* = 131) belonged to phylogroup B2. Of the 725 human-source *E. coli* isolates, 127 were found to belong to ST95 (17.5%), while 62 of the 493 avian-source *E. coli* isolates belonged to ST95 (12.6%). These strains were further genotyped by MLST. All ST95 isolates identified by MLST were identical to the strains that yielded four products with the multiplex PCR. The multiplex PCR method achieved a specificity of 100% and a sensitivity of 100% for the detection of ST95. When compared with MLST and gene-sequencing methods, this multiplex PCR method was more convenient and was able to directly identify ST95 strains in clinical *E. coli* isolates.

## 4. Discussion

Genomic surveillance using whole-genome sequencing (WGS) offers high-resolution analysis of bacterial population diversity, persistence, and evolution. It can also be used to report the presence of virulence and resistance genes [52,53]. The ST95 APEC/ExPEC strain is a significant extraintestinal pathogen and is well-documented in connection with poultry diseases and human extraintestinal illness, such as UTIs and bloodstream infections. It was identified as the largest clonal group among *E. coli* isolates collected from patients with UTIs in California, during 1999 to 2000 and 2016 to 2017 [54]. However, to date, WGS studies on ST95 have been limited. In this study, we performed an in-depth retrospective analysis of WGS data from 1081 ST95 isolates collected from the NCBI and Enterobase databases. Our study formed part of a broader global endeavor to understand the pathogenic ST95 clone. The pan-genome of ST95 strains was estimated to be in excess of 30,000 genes, which indicated the massive diversity of ST95. Previous studies on the size of the ST95 pan-genome have been significantly smaller, as have the datasets used to conduct the research. An investigation by David M et al. analyzed the pan-genome of 200 ST95 isolates from various origins and identified 17,603 genes, of which 3134 belonged to the core genome [55]. Therefore, the pan-genome will continue to enlarge with the inclusion of more whole genome sequences. Full sequencing of an *E. coli* strain permits just one-fourth of the pan-genome to be observed. This means that, while a model strain may be used to study some fundamental processes, no one strain can be considered as perfect.

It has long been recognized that there are genetic substructures in ST95 strains [56]. Our phylogenetic analysis showed a clearly defined five-group population structure of ST95. Structures similar to ours have also been reported in a recent study performed by Gordon et al. [55], which designated the five main clusters as being related to serotypes and *fimH* alleles. In our study, in addition to the prevalent *fimH* alleles (*fimH* 15, 27, 30, 41, and 54), we also found alleles such as *fimH*483, *fimH*526, and other serotypes, which included O6:H7. Clades Ⅰ and Ⅱ were found to have a high diversity of *fimH* alleles. As the number of ST95 strains increases, the diversity of *fimH* alleles will also increase. It can be inferred that *fimH* allele variation in ST95 isolates may reveal the molecular mechanisms that underlie clonal diversification. Scott J et al. analyzed molecular variation in *fimH* and revealed that *E. coli* strains that colonize different niches benefit from structural SNPs in *fimH,* which are under significant positive selection for pathoadaptive functional change [57]. Our study also took advantage of the wealth of information available in the accessory genome to improve the resolution of this population structure.

There is little information available about the evolution and emergence of ST95 strains. In our study, we used BEAST to analyze this. The ST95 strain can be considered to be an ancient and long-standing lineage, with a long period of evolution. A Bayesian skyline analysis of ST95 demonstrated multiple sequential increases in population size, which began in the early 1840s. The population size then leveled out after the 1970s. In contrast, ST131 had a short period of evolution, but its population size has expanded rapidly over the last decade. This strain, which is famous for its multidrug-resistant phenotype, is a relatively young ST clone: the most recent common ancestor for ST131 can be dated to approximately 140 years ago [58]. Several studies have revealed that the evolution of ST131 has been driven by antibiotics. Nicole et al. reported that the global development of drug-resistant clades (C1/H30 and C2/H30Rx) began approximately 25 years ago, which is concordant with the intensive selection pressure created by the wide range of use of extended-spectrum cephalosporins and fluoroquinolones [59]. This indicates that persistent selection pressures exerted by antimicrobials can drive the selection and emergence of MDR ExPEC lineages, such as ST69. However, we noticed that ST95 may be less influenced by selection pressures from antimicrobials, although antibiotic resistance has been found to increase over time. Therefore, ST95 and ST131 may have distinct evolutionary paths. The evolution of ST95 may be influenced and shaped by multiple factors, possibly related to selection and expansion in the intestinal reservoir of the host. Drug resistance does not appear to be the primary driving force for the evolution of ST95, which may have been driven by pathogenicity. Our study found that ST95 existed in hosts long before the use of antibiotics and evolved in parallel in humans and poultry over a long period of time. The ST95 strain may have undergone an intrinsic biological adaptation, at the species level, over vast periods of time, and it has evolved a series of factors to adapt to the host environment. The ST95 strains that were recovered from different hosts and geographic sources were randomly located on the tree, which suggested no host specificity for ST95 isolates. As a traditional ExPEC lineage, ST95 will not be substituted by an emerging lineage, and it was found that after the introduction of new lineages, ST131, ST69 and ST95 still maintained a relatively stable population [60]. The ST95 and ST131 strains are major pandemic ExPEC lineages, but there are significant differences in their sensitivity characteristics. This indicates that drug resistance does not seem to be the primary driving force responsible for the success or prevalence of *E. coli* lineages. In our study, multiple VFs, from different functional categories, were detected frequently in ST95. These VFs contributed to the adaptations, persistence, and strong competitiveness of ExPECs in the host internal environment and during environmental dissemination.

Whole-genome sequencing enables researchers to examine the presence and absence of genes in clinical isolates to provide information on the significance of virulence genes in pathogenesis. Our analysis showed that ST95 strains possess a considerable number of virulence genes. We discovered a significant frequency of genes linked to ExPEC or APEC virulence, which indicated that ST95 strains have a high pathogenic potential. The isolates that belonged to ST95 showed a higher mean VF-encoding gene score than other pandemic STs [61,62]. Our findings supported the view that the high number of VF genes in ST95 strains may be part of what facilitates the success of ST95. In addition, the presence of the ColV/BM plasmid is a defining feature of the APEC pathotype [63,64], and some virulence genes that are associated with this plasmid were also found at a high frequency in human ST95. This indicated that zoonotic dissemination of APEC can occur from poultry to humans. Plasmid typing analysis revealed that the plasmid replicon types found in ST95 strains were highly diverse, and there were multiple combinations of types. The IncFII and IncFIB plasmid replicons were abundant in ST95 isolates, and plasmids that carried multiple virulence genes were identified. However, plasmids were scattered across the isolates, and there was no strong plasmid–clade adaptation. We hope that future studies will provide better insight into the molecular mechanisms by which large plasmids promote the pathogenesis and evolution of ST95. Interestingly, there were variations in virulence profiles, some of which were related to the population structure of different clades. Several genes (*ibeA*, *sfaS*/*focD*, *cnf1*, *cnf2*, and *hlyA/D*) were almost exclusively found in the Clade Ⅴ strains, which indicated an increase in the virulence potential. We also identified that PAI-I 536 and GimA were exclusively present in Clade Ⅴ. The acquisition of PAIs, through horizontal transfer, may have a significant impact on the evolution of ST95 to form new clades or lineages.

The ST95 lineage has been commonly described as a drug-sensitive lineage that is devoid of plasmids [44]. However, it was evident in our study that the large collection of isolates had a higher overall prevalence of resistance and multi-drug resistance than reported in previous studies. We detected the presence of several *β*-lactamase genes, such as *bla*_TEM-1_, *bla*_CTX-1_, *bla*_OXA-1_, *bla*_KPC-1_, and *bla*_CMY-1_, which confer resistance to most *β*-lactam antimicrobials in ST95 strains. The antimicrobial resistance genes associated with sulfonamide, aminoglycoside, tetracycline, and streptomycin were also detected. The ST95 strain is considered to be a globally important cause of extraintestinal *E. coli* infections. The emergence of ST95 strains with high levels of resistance and an extensive virulence profile is of great concern, as they can result in a high risk of treatment failure. Further studies are needed to determine whether this multi-drug resistant phenotype will spread quickly through ST95 populations over time.

In this study, we established a novel, rapid PCR method for the detection of ST95. The four genes targeted by this multiplex PCR are conserved in ST95 genomes but are rare or absent in the other STs. This method proved to be 100% sensitive and specific in screening ST95 strains from human- or poultry-source *E. coli* isolates. The assay allows any laboratory that is equipped for PCR to correctly, rapidly, and cost-effectively distinguish ST95 from other strains. It can also be used in combination with published assays associated with the PCR detection of other STs, such as ST131 and ST648 [65,66]. This method simplifies screening for the dominant STs in large *E. coli* populations, which is vital for epidemiological studies and monitoring of clonal trends. In the future, this unique PCR method can also be applied to establish PCR assays for the identification of other STs.

## Figures and Tables

**Table 1:** Primer sequences and sizes of PCR products used in the ST95 multiplex PCR assay.

Primer	Primer Sequence	Length (nt)	Tm (°C)	% GC	Amplicon Size (bp)
p-sfmH _For CTCCAGAAAACAATACCACACC		22	50.5	45.5	996bp
p-sfmH _Rev ATTCCACTCGCAATATAGCCAG		22	52.9	45.5	
group4861-F TACAACAGTTTCCTCCAGCC		20	49.2	50	474bp
group4861-R CTTTAACCATGACATCCCAG		20	46.9	45	
group 8607-F TCTTGTTCGTTGTTTTTATCGTCGG		25	58.3	40%	775bp
group 8607-R TTTTATCACGCCAGGTGAAGAGTGA		25	58.8	44%	
group9754-F ACAAAATGGCTAAAAAGGAGTGATG		25	54.9	36%	256bp
group9754-R CCACCGACTTGTAATTCCTCTAACA		25	55.5	44%	

## Data Availability

All data generated or analyzed during this study are included in this published article.

## Supplementary Materials

The following supporting information can be downloaded at: https://www.mdpi.com/article/10.3390/pathogens11121489/s1, Figure S1. Time-scale Bayesian phylogenetic tree with the detailed information of ST95 E. coli strains. Table S1. Detailed information (collected time, diverse hosts, and various countries) on the 1081 publicly available whole genomes of ST95 ExPEC; Table S2. Information on structure population for five clades (Clade Ⅰ to Clade Ⅴ) and 27 clusters based on accessory genes in ST95 ExPEC; Table S3. Carriages of virulence genes, resistance genes, and plasmid replicons in ExPEC ST95; Table S4. The plasmid modules predicted in ST95 ExPEC strains; Table S5. The distribution of pathogenicity islands (PAIs) among ST95 ExPEC strains; Table S6. The unique genes identified in the ST95 pan-genome compared with the genes in other ST pan-genomes; Table S7. The specific genes in the ST95 pan-genome selected by the reads maps of gut metagenomes.
