# Supplementary figures and images for: Genomics Analysis to Identify Multiple Genetic Determinants That Drive the Global Transmission of the Pandemic ST95 Lineage of Extraintestinal Pathogenic Escherichia coli (ExPEC)
Source: Pathogens. 2022 Dec 7;11(12):1489. doi: 10.3390/pathogens11121489 (PMC9781279; doi:10.3390/pathogens11121489)

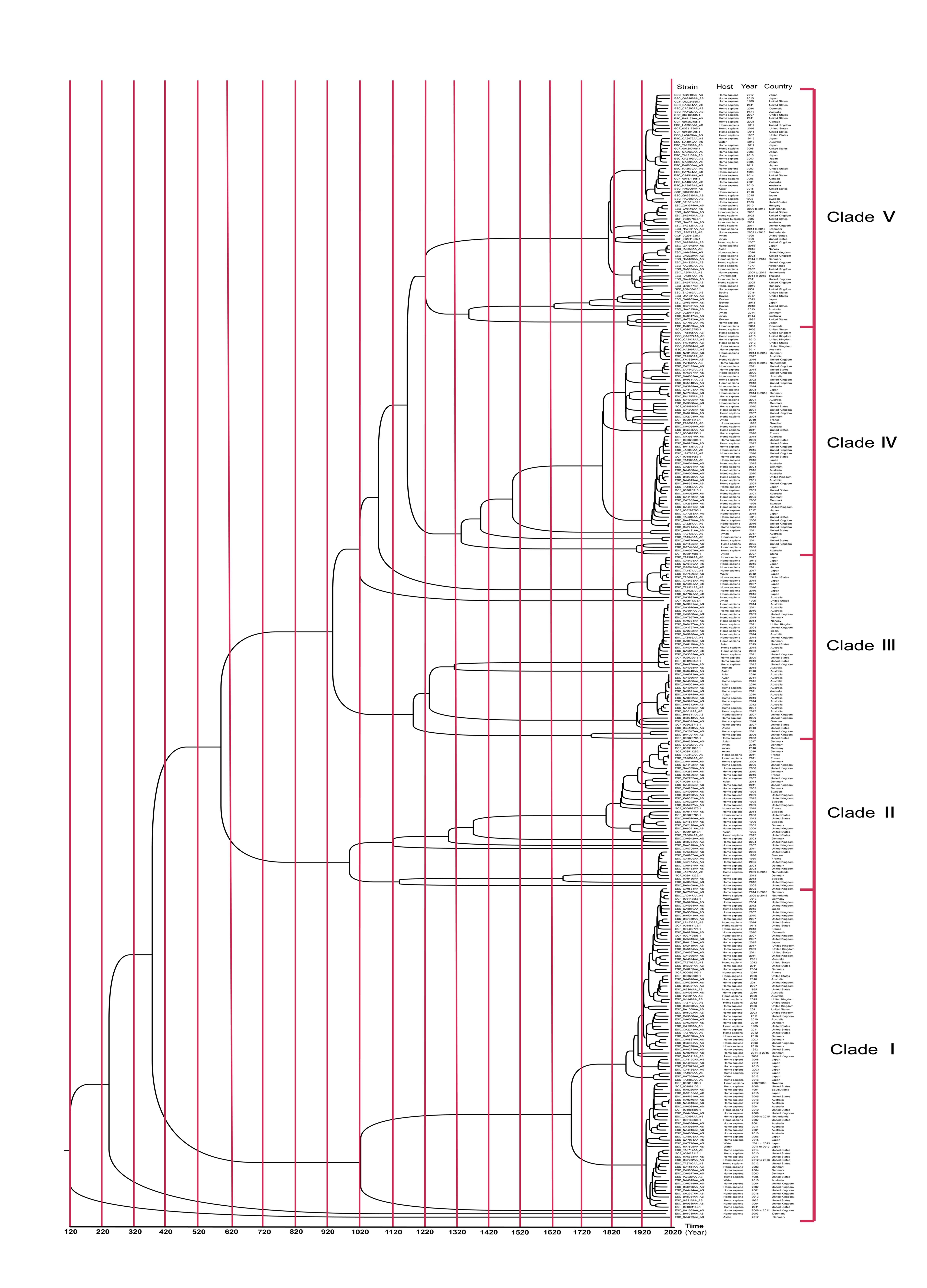

Supplement: Supplementary file 1 [file pathogens-11-01489-s001.zip › Supplementary Materials/Figure S1.jpg]
